# Ten-electron count rule for the binding of adsorbates on single-atom alloy catalysts

**DOI:** 10.1038/s41557-023-01424-6

**Published:** 2024-01-23

**Authors:** Julia Schumann, Michail Stamatakis, Angelos Michaelides, Romain Réocreux

**Affiliations:** 1 Thomas Young Centre and Department of Chemical Engineering, University College London, London, UK; 2 Yusuf Hamied Department of Chemistry, University of Cambridge, Cambridge, UK; 3 Physics Department and IRIS Adlershof, Humboldt Universität zu Berlin, Berlin, Germany; 4 Department of Chemistry, University of Oxford, Oxford, UK

**Keywords:** Chemical physics, Density functional theory, Heterogeneous catalysis

## Abstract

Single-atom alloys have recently emerged as highly active and selective alloy catalysts. Unlike pure metals, single-atom alloys escape the well-established conceptual framework developed nearly three decades ago for predicting catalytic performance. Although this offers the opportunity to explore so far unattainable chemistries, this leaves us without a simple guide for the design of single-atom alloys able to catalyse targeted reactions. Here, based on thousands of density functional theory calculations, we reveal a 10-electron count rule for the binding of adsorbates on the dopant atoms, usually the active sites, of single-atom alloy surfaces. A simple molecular orbital approach rationalizes this rule and the nature of the adsorbate–dopant interaction. In addition, our intuitive model can accelerate the rational design of single-atom alloy catalysts. Indeed, we illustrate how the unique insights provided by the electron count rule help identify the most promising dopant for an industrially relevant hydrogenation reaction, thereby reducing the number of potential materials by more than one order of magnitude.

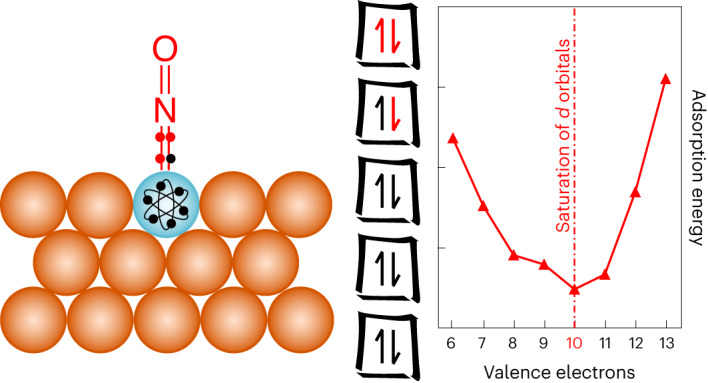

## Abstract (web summary)

Single-atom alloys have emerged as highly active and selective catalysts that do not follow the traditional models of heterogeneous catalysis. Now it has been shown that the binding of adsorbates at their surface abides by a simple 10-electron count rule, which can identify promising catalysts for various applications.

## Main

Single-atom alloys (SAAs) have recently emerged as a new class of catalysts able to reach high activity and selectivity for a range of chemical reactions^1–3^. In these alloys, the active metal is dispersed as single atoms at the surface of a more inert host metal. This doping strategy significantly improves the catalytic performance of the otherwise poorly reactive, and yet highly selective, coinage metals (Cu, Ag and Au). There has been considerable work aiming at screening and predicting the unique reactivity and catalytic performance of SAAs^2,4,5^. Despite the accuracy of the predictions based on density functional theory (DFT), a simple physical model describing general trends is still lacking due to the special electronic structure of SAAs^1,2^.

Traditionally, the catalytic activity of a material can be described using the binding energy of species involved in the mechanism of the catalysed reaction. According to the Sabatier principle, species should bind neither too weakly nor too strongly to ensure optimal catalytic activity^6^, and the electronic properties of the catalyst determine the binding energies. For transition metal catalysts, the adsorption energy of a species linearly correlates with the energy centre of the electronic band consisting of the *d*-states of the metal^7,8^. This means that the more valence electrons the metal has, the lower the energy of the *d*-band centre and the weaker the bond to an adsorbate at the surface (Supplementary Fig. 1). Although the *d*-band model has shown great success in understanding catalytic performance over a few decades, there are known limitations. For example, SAAs and the related class of near-surface alloys (NSAs) can both escape the trends expected from the *d*-band model^9–12^. Recent developments have shown that NSAs follow stability rules and require corrections to the *d*-band model to fully describe their properties^9,13^. Yet, such an approach is not applicable to SAAs. Alternatively, the rise of machine learning has provided an efficient approach for the prediction of adsorption energies on traditional alloys and SAAs^5,14–16^. Albeit faster than and reportedly as accurate as DFT calculations, these models do not provide the fundamental physical principles that govern the stability of adsorbates on SAA surfaces. In a change of perspective, a few studies have suggested to consider SAAs as analogues of molecular systems^17–20^. The stability of such systems is related to the filling of discrete states (atomic orbitals or molecular orbitals (MO)) resulting in various electron-counting rules (octet, 4*n* + 2 aromaticity rule and so on)^21–23^.

Here, we propose a change of paradigm in the way we rationalize the reactivity of SAAs. Moving away from the traditional linear scaling relationships that have limited applicability for SAAs, we demonstrate that an MO approach, albeit somewhat simplistic, provides profound insight into the metal–adsorbate binding mechanism. Screening a variety of catalytically relevant adsorbates on a large set of SAA surfaces, we show that the adsorbate–dopant interaction is strongest when the *v*_M_ valence electrons of the metal dopant (equivalently its group number in the periodic table) and the *k* valence electrons of the adsorbates interacting with the dopant sum up to ten: *v*_M_ + *k* = 10. This 10-electron count rule is supported by a detailed analysis of the electronic structure of adsorbates bound to SAAs surfaces and generalized to molecular adsorbates. Finally, we demonstrate that, without expensive DFT calculations or complex machine learning models, this rule provides, experimentalists and theoreticians alike, insightful guidance for the design of SAA catalysts for targeted reactions of industrial significance, as illustrated herein for the reduction of nitrogen.

## Results and discussion

### Adsorption of adatoms

**Fig. 1:**
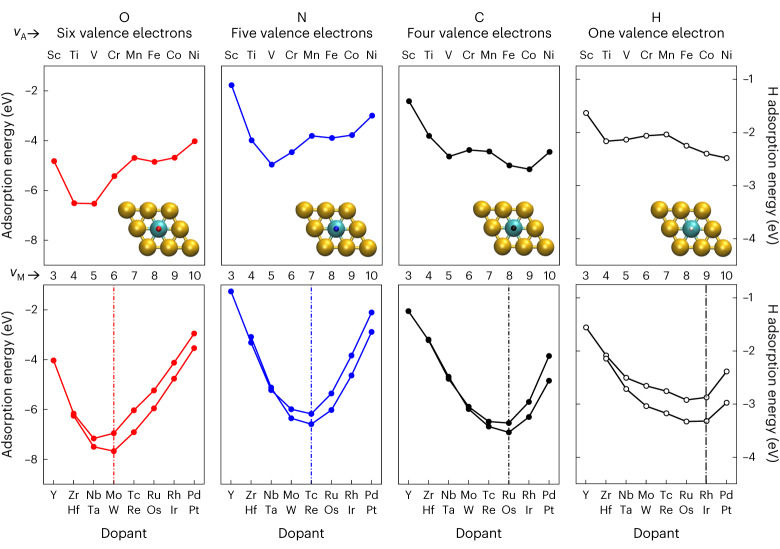
Periodic trends for the binding energies of atomic adsorbates (O, N, C, H) on Au-based SAA surfaces doped with transition metal single atoms. Top, the trends for 3*d* dopants show a fixed maximum in the middle of the period (close to Mn) whereas, bottom, the trends for 4*d* and 5*d* dopants show minima that shift depending on the number of valence electrons of the adsorbates. The number of valence electrons of the dopant (*v*_M_) is reported as a secondary *x*-axis, and the approximate position of the minimum is highlighted by a vertical dashed dotted line. The inserts show the unit cell used to compute the adsorption energy of the adsorbate in the atop position on the dopant. Results for the Cu and Ag hosts are shown in Supplementary Fig. 2. Source data

We start by analysing the periodic trends in the adsorption energies of key atomic adsorbates. Specifically, in Fig. 1 and Supplementary Fig. 2, we show the adsorption energies computed with DFT for atoms (O, N, C and H) adsorbed on a whole range of transition metal dopants on SAA surfaces. Unlike the adsorption on pure transition metals^8,24^, we do not observe the monotonic weakening of the adsorbates’ binding from left to right along the periodic table (Supplementary Fig. 1a,b), despite similar trends of the centre of the *d*-states of the dopants (Supplementary Fig. 3). Instead, we observe shallow W-shaped trends for adsorption energies on 3*d* metal dopants and deep V-shaped trends for adsorption energies on 4*d* and 5*d* dopants. Interestingly, the position of the minimum on 4*d* and 5*d* dopants depends on the adsorbate, but not the dopant’s period nor the host material. When the number of valence electrons of the adsorbate (*v*_A_) decreases (O > N > C > H), the minimum shifts to metal dopants with more valence electrons *v*_M_ (to the right of the period), as if the dopant had to compensate for the fewer electrons brought by the adsorbate.

To further analyse the interplay between the number of electrons brought by the adsorbate and the dopant, we can tentatively count the total number of valence electrons of the dopant–adsorbate system, ignoring the host metal. For the dopants, we consider the number of electrons of the *s-* and *d-*orbitals of the outer shell, which corresponds to the group number of the element. For adsorbates, we consider all the electrons of the *nsnp* outer shell: these are the electrons traditionally considered when drawing Lewis structures. Now, if we add the valence electrons of the dopant and the adsorbate, we find that strongest binding is associated with 10 electrons for H and 12 electrons for *p*-block adsorbates (O, N and C). For 3*d* dopants, we can still recognize a preference towards binding to early transition metals for electron-rich adsorbates (O and N), and to later transition metals for adsorbates with fewer electrons (H and C). However, because of significant spin effects on magnetic 3*d* dopants, adsorption is weakened in the middle of the row, with a fixed extremum for Mn for all adsorbates. Indeed, if the effect of spin is unrealistically suppressed, 3*d* dopants behave like 4*d* and 5*d* dopants (Supplementary Fig. 4). Interestingly, these binding features on SAA surfaces (shape of the trends, role of the number of valence electrons) are distinct from pure transition metal surfaces but analogous to the binding of ligands in organometallic complexes (as shown in Supplementary Fig. 1) successfully modelled using an MO approach^23^.

**Fig. 2:**
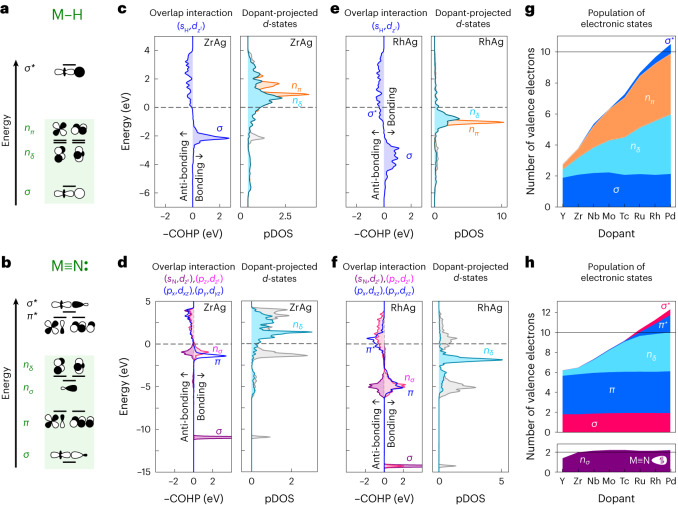
Comparison between the electronic states of H and N bound to transition metals in dinuclear complexes and on SAA surfaces. **a**,**b**, MO diagrams for the dinuclear complexes MH (**a**) and MN (**b**), where M represents a transition metal. **c**–**f**, COHP analysis and pDOS for H (**c**,**e**) and N (**d**,**f**) on Ag surfaces doped with Zr (**c**,**d**), and Rh (**e**,**f**). These analyses show well-defined electronic states akin to the MOs of dinuclear complexes with similar ordering and filling. The dashed lines show the Fermi level, that is, the energy cut-off between populated states (below the line) and nonpopulated states (above the line). **g**,**h**, Full population analysis of the electronic states of H (**g**) and N (**h**) adsorbed on SAA surfaces. Population of antibonding states becomes significant when the five orbitals with *d*-contributions are saturated with ten electrons. Source data

This analogy with organometallic complexes and the electronic structure of SAAs akin to gas phase atoms^17,18^ have motivated us to consider an MO approach to rationalize the binding of adsorbates on SAAs (Fig. 2). Based on the symmetry point group *C*_∞ν_ of the metal–adsorbate pair, we can construct an MO diagram (Supplementary Fig. 5). For H, the *s-*orbital can interact with the metal’s *d*_*z*²_ orbital, generating a bonding *σ* and an antibonding σ* MO (Fig. 2a). The remaining four *d*-orbitals form the nonbonding *n*_*δ*_ and *n*_*π*_ MOs. We can therefore fill up to five MOs before populating the antibonding σ*-orbital and thereby weakening the adsorbate–metal bond (Fig. 2a). Similarly, we can construct the MO diagrams for C, N and O. These adsorbates have partially filled *p*-orbitals that can contribute to the bond. The linear combination of the metal *d*_*z*²_ and the adsorbate’s *s* and *p*_*z*_ orbitals generate a bonding *σ* MO, a nonbonding *n*_*σ*_ MO and an antibonding σ* MO. Additionally, the linear combination of *d*_*xz*_ and *d*_*yz*_ with the *p*_*x*_ and *p*_*y*_, form two bonding *π* and two antibonding π* MOs. The remaining two *d*-orbitals, which cannot interact with the adsorbate’s orbitals, form the nonbonding *n*_*δ*_ MOs. For *p*-block adsorbates, we can fill up to six MOs, that is, twelve electrons, before populating antibonding orbitals (Fig. 2e). This MO approach seems to accurately predict the point when the dopant–adsorbate’s binding weakens. This is under the assumption that the dopant’s *s*-orbital can be taken out of the picture and its electron(s) populate the states that we have just built.

To ensure that our MO approach appropriately captures the binding of adsorbates on SAAs (position of the orbitals and population), we further analysed the electronic structure of H (Fig. 2c,e,g) and N (Fig. 2d,f,h) adsorbed on Ag-based SAAs. When constructing MO diagrams, we are interested in knowing (1) where the MOs are on the energy scale (*y*-axis), and (2) which atomic orbitals interact to form a particular MO (that is, atomic orbitals with nonzero overlap interaction). This information can be extracted, from DFT calculations, using the electronic density of states (DOS), that is, the number of states found at a certain energy level, projected onto the *d*-states of the dopant (pDOS), and the crystal orbital Hamilton population (COHP) that quantifies the overlap interaction between two orbitals^25,26^. Fig. 2c shows the COHP analysis between the *d*_*z*²_ orbital of Zr and the *s*-orbital of H as extracted from the electronic structure calculation of H adsorbed on ZrAg. The analysis shows a narrow positive peak at −2 eV for the pairwise interaction between the *s* and *d*_*z*²_ orbitals; this is the expected bonding *σ* MO. The same plot shows antibonding σ* states as a negative broad band. The four other *d*-orbitals, referred to as *n*_*π*_ and *n*_*δ*_ in Fig. 2a, do not interact with any orbitals of H, and thus cannot be identified in the COHP analysis that only shows pairwise interactions. Instead, they appear in the plot showing the density of *n*_*π*_ and *n*_*δ*_ states (Fig. 2c, right) as partially populated, as expected from the MO diagram. When considering H adsorbed on RhAg (Fig. 2e), all the states shift to lower energies, with the *σ*, *n*_*π*_ and *n*_*δ*_ orbitals now being doubly occupied and the σ* partially occupied. This results from Rh having more electrons than Zr. The same analysis for adsorbed N is shown in Fig. 2d,f,h. The COHP analysis shows that the *σ*-orbital indeed results from the interaction of the *d*_*z*²_ orbital with the adsorbate’s *s* and *p*_*z*_ orbitals. Approximately 7 eV higher up in energy are found the *π* orbitals (blue curve). In the same energy region, the COHP analysis shows that the *d*_*z*²_ orbital interacts again with the adsorbate’s *s* and *p*_*z*_ orbitals. This time, however, the binding contribution of the *p*_*z*_–*d*_*z*²_ interaction (pink curve) cancels out the antibonding contribution of the *s*–*d*_*z*²_ interaction: the superposition of these two opposite contributions corresponds to the expected nonbonding *n*_*σ*_ MO. Overall, the proposed MO diagrams agree well with the computed electronic structures. Now that we have identified the different orbitals, we can estimate their filling. PdH (Fig. 2g) and RhN (Fig. 2h) have more than 10 and 12 valence electrons, respectively (*v*_A_ + *v*_M_). Although the dopant’s *s* states—which interact with the host’s *s* states to form a band—are half-filled (Supplementary Table 1), there are more than *v*_M_ − 1 electrons in the orbitals considered in Fig. 2. This can be attributed to charge transfer between the host and the dopant resulting in more electrons than perhaps expected, especially around the point of strongest binding and beyond. All the valence electrons of the metal (*v*_M_) should therefore be counted to predict when antibonding states start being populated. This whole analysis also provides insights into the binding of adsorbates on 3*d* metals. The electronic structure of N adsorbed on 3*d* dopants shows that the filling of antibonding states starts as early as Cr, the dopant for which the binding shows signs of weakening (Fig. 1) and intensifies for Co and Ni (Supplementary Fig. 6). This is consistent with the observed W-shape trend of the adsorption energies for 3*d* dopants and our proposed analogy to organometallic complexes.

The common point between H and *p*-block elements adsorbed on SAAs is that adsorption is the strongest when all the bonding and nonbonding MOs with *d* contributions are filled (Fig. 2g,h). The adsorption energy starts weakening when antibonding states are being populated. But why does filling nonbonding states seemingly strengthen the bond? Nonbonding states do not contribute to increased stabilization. What drives the stabilization is the lowering of the dopant’s levels and the reduction of the dopant’s radius which, together, lead to a better overlap with the adsorbate’s small orbitals at shorter distances (Supplementary Fig. 7). Now, if H and *p*-block elements seem to obey different counting rules, it is only because too many electrons are counted in the latter case. The *n*_*σ*_ orbital, which can be interpreted as a lone pair located on the adsorbate outside the internuclear region, is consistently populated throughout the period and does not contribute to the binding or the saturation of the *d* orbitals. Ignoring this lone pair results in a universal 10-electron count rule for maximal binding of adsorbates on 4*d* and 5*d* dopants (Fig. 2g,h). It is essential to distinguish between the adsorbate’s total number of valence electrons *v*_A_ and the number of valence electrons *k* that interact with the *d* states, especially when considering larger adsorbates such as molecules (Table 1). For example, CO has *v*_A_ = 10 valence electrons, but, as expected from organometallic chemistry, only *k* = 2 electrons interact with the transition metal^23,27^. With this distinction in mind, the dopants with maximal binding to a specific adsorbate are easily identified as those with 10 − *k* valence electrons, hereafter referred to as *d*^10−*k*^ dopants.

**Table 1:** Total number of valence electrons (*v*_A_) and number of valence electrons interacting with the metal dopant (*k*) for atomic and molecular adsorbates

	H	C	N	O	H_2_O	NH_3_	CO	N_2_	Linear NO	Bent NO	C_2_H_4_
*v*_A_	1	4	5	6	8	8	10	10	11	11	12
*k*	1	2	3	4	2	2	2	2	3	1	2

### Extension to molecular fragments

**Fig. 3:**
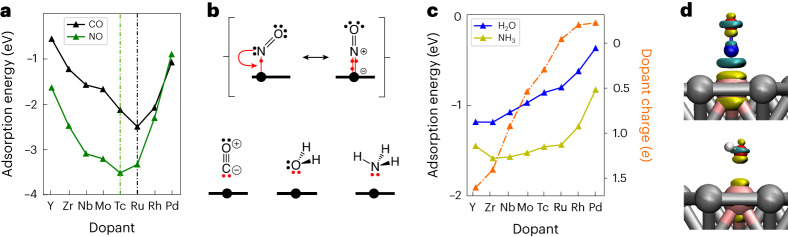
Adsorption of molecules on SAA surfaces. **a**, Adsorption energy of CO and NO showing V-shaped trends with minima for Tc (*d*^7^) and Ru (*d*^8^), respectively. **b**, Lewis structures of CO and NO (including mesomerism) showing that the number of electrons directly interacting with the dopant (red dots near the larger black ball representing the dopant) is consistent with the 10-electron count rule. **c**, Despite H_2_O and NH_3_ having the same number of electrons directly interacting with the dopant as CO, the adsorption energies do not show minima. This is attributed to the electrostatic contributions that dominate the trend in this specific case (larger adsorption energies for more positively charged dopants). **d**, Electronic density difference for NO (upper panel) and H_2_O (lower panel) indicating charge depletion (yellow) and charge accumulation (cyan) upon adsorption on RhAg SAA (threshold of ±0.06 e·Å^−3^). Source data

We have shown the 10-electron count rule for the stability of atomic adsorbates on SAAs. Now, we extend this rule to molecular adsorbates. For example, CO interacts with its lone pair located on the carbon atom (Fig. 3b). NO, as a radical, can either interact with one electron in a bent geometry or three electrons in a linear geometry (Fig. 3b). If we only consider the linear geometry, the 10-electron rule predicts the strongest adsorption energies at *d*^8^ for CO and *d*^7^ for NO on 4*d* and 5*d* dopants. This is confirmed by our DFT calculations (Fig. 3a). It is important to note, however, that this rule only holds when the binding trends between the adsorbate and the dopant are dominated by the sharing of electrons, that is, covalent contributions. H_2_O and NH_3_ are known examples of adsorbates for which the binding trends are dominated by electrostatic interactions^20^. At the electronic level, this translates to a limited reshuffling of the electronic density of H_2_O upon adsorption compared with NO (Fig. 3d). For these adsorbates, the DFT-computed adsorption energies do not show the expected minima and roughly follow the steady variation of the electric charge of the dopant (Fig. 3c and Supplementary Table 2) over the 4*d* period (from **−**0.22*e* to +1.61*e*). It is only when we remove the electrostatic contribution, that we recover the expected trends for the covalent contribution with minima at *d*^8^ for NH_3_ and H_2_O (Supplementary Fig. 8). For these closed-shell molecules with highly polarized bonds (large electronegativity difference between O or N and H) as well as halogens and hydroxyl (Supplementary Fig. 9), the 10-electron count rule has less practical applicability and the atomic charge of the dopant is a much more robust descriptor of the binding^20^.

**Fig. 4:**
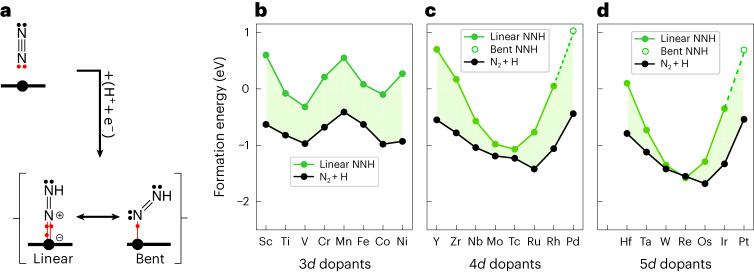
Hydrogenation of nitrogen to diazenyl NNH. **a**, Lewis structures and different binding modes of NNH. **b**–**d**, Formation energy of N_2_ and NNH (in different geometries) on Au-based SAAs considering 3*d* (**b**), 4*d* (**c**) and 5*d* (**d**) dopants. The shaded green area indicates the reaction energy, that is, the endothermicity of the hydrogenation of N_2_ to NNH. By minimizing the shaded area, the reaction gets thermodynamically more favourable. Energies are referenced with respect to N_2_ (g) and H adsorbed on Au. Source data

The significance of the 10-electron rule goes beyond understanding periodic trends in binding on SAAs: it helps identify active catalysts for targeted reactions. Let us consider the reduction of nitrogen to ammonia, a reaction of industrial relevance. On Au-based SAAs, the first elementary step, namely the hydrogenation of N_2_ to diazenyl NNH (Fig. 4a), was identified as rate-determining because of its endothermicity^28^. N_2_ interacts with the dopant via its lone pair and is predicted by the 10-electron count rule to have strongest binding for *d*^8^ (Ru, Os) on 4*d* and 5*d* dopants (Fig. 4c,d, black line). NNH, isolobal to NO, is predicted to have the strongest adsorption for *d*^7^ (Tc, Re) on 4d and 5*d* dopants. DFT calculations, shown in Fig. 4c,d, again confirm these predictions. Now, let us consider the trends in reaction energy required to go from the reactant (black curve) to the intermediate (green curve). Because of the rigidity of the periodic trends on 3*d* doped surfaces (W-shape with fixed maximum for Mn), the reaction energy does not significantly change when screening 3*d* dopants. However, for 4*d* and 5*d* dopants, the situation is different. Because of the curvatures and the different position of adsorption minima for N_2_ and NNH, the gap between the two curves closes when the binding energy of NNH is the strongest. We therefore predict the first hydrogenation of N_2_ to be most facile on *d*^7^ dopants, that is, Re and Tc. For reasons ranging from synthesizability, cost, to stability (Tc is not a stable isotope, it is only considered to test and illustrate the counting rule), it is worth considering dopants around the *d*^7^ minimum. Moving to the left of the periodic table (*d*^7^ to *d*^6^) provides viable options (Mo and W) with small thermodynamic barriers. Moving to the right (*d*^7^ to *d*^8^), however, is less interesting as the stability of the reactant N_2_ reaches its maximum for *d*^8^, thereby detrimentally increasing the reaction energy.

Interestingly, previous computationally demanding high-throughput and machine-learning studies^28,29^ had identified similar SAAs for the reduction of N_2_. According to a previous study, Re dopants offer the best compromise between donation and back-donation to weaken the N≡N bond^29^. Our theoretical framework offers an alternative explanation: this is the stability of NNH that controls the choice of the dopant regarding the most favourable reaction energetics, although donation and backdonation may, admittedly, play a role for the scission of the bond between the two nitrogen atoms in the subsequent steps of the reduction mechanism. Of course, this analysis is an early step towards the demonstration of a new catalyst for ammonia synthesis. However, this example illustrates the power of our approach: the counting rule remarkably identifies, without access to supercomputers, the most promising catalytic sites, and the degree to which we can extend the search of efficient catalysts to neighbouring dopants. Once identified, computational effort (multiscale modelling including effects such as solvation, electric fields, kinetics) or experimental effort can be specifically directed to the most promising materials.

In the previous paragraph we have only focused on the linear geometry of NNH, which is the most stable in the region of catalytic interest. We have identified two other geometries: the flat-lying and the bent geometries. The former binds to the dopant bringing five electrons and therefore helps early transition metals to satisfy the 10-electron rule, especially for dopants with fewer than 5 valence electrons. The bent geometry, interacting with one electron, is the only geometry found for Pd and Pt dopants. For these late transition metals, the bent geometry, with fewer electrons interacting with the *d* orbitals of the dopants, prevents too many antibonding states from being filled. The preference of early, mid and late transition metals for the flat-lying, linear and bent geometries is again a manifestation of the 10-electron count rule (Supplementary Fig. 10). Similar behaviour is well known in molecular and organometallic chemistry and was theorized using orbital correlation diagrams^20,30,31^.

## Conclusion

In summary, species covalently bound at the surface of SAAs show non-monotonic binding energy trends that mostly depend on the nature of the dopant site. Our work clearly identifies the dopants with stronger or weaker affinity for a given adsorbate. For 3*d* dopants, binding is the strongest on Ti and V, or Fe and Co, regardless of the nature of the adsorbate. For 4*d* and 5*d* dopants, the trends show a single extremum with strongest binding when the adsorbate’s electrons fill and saturate the dopant’s *d* orbitals, hence the 10-electron count rule. In this case, the dopants with strongest binding depend on the nature of the adsorbate. We show that a simple molecular orbital approach rationalizes these rules. This furthers our previous work that had successfully identified the dopant charge as a descriptor for the binding of species with large electrostatic contributions but had originally failed at providing an electronic-level descriptor for the covalent contribution^20^. The fundamental principles identified here advance our understanding of chemical bonds on SAAs and provide a sought-after alternative to the *d*-band model for binding trends on the more reactive dopant sites (the *d*-band model still remains relevant for host sites). Finally, our model bridges the gap between the concepts of heterogeneous and homogeneous catalysis and, significantly so, establishes a clear conceptual guide, for theoreticians and experimentalists alike, for the design of more efficient catalysts, without the expensive or complex simulations only accessible to computational scientists.

## Methods

All DFT calculations were performed using Vienna Ab Initio Simulation Package (VASP) version 5.4.4 (refs. ^32,33^) with the projector-augmented wave^34^ method to model core ionic potentials. The nonlocal optB86b-vdW exchange-correlation functional^35,36^ was used for all electronic structure calculations except the systems with CO and NO adsorption. The plane wave cut-off was set to 400 eV, electronic convergence set to 10^−6^ eV and geometric convergence criterion was less than 0.02 eV Å^−1^. The surfaces were modelled using a five-layer *p*(3×3) slab, with the bottom two layers fixed at the positions of the bulk host. The pure fcc host metals were optimized in bulk and lattice constants of 3.60 Å for Cu-based SAAs, 4.09 Å for Ag-based SAAs and 4.13 Å for Au-based SAAs were used. The slabs were separated by a 15 Å vacuum layer. For the screening of adsorption energies, a 3×3×1 Monkhorst−Pack^37^ *k*-point mesh was chosen for Brillouin-zone integration, which was shown to yield the same trends as a fully converged 13×13×1 Monkhorst-Pack *k*-point mesh (Supplementary Fig. 11). All adsorbates were placed at the atop position and optimized using the quasi-Newton algorithm implemented in VASP, to prevent relaxation of some adsorbates to the fcc site. Overall trends are preserved for fcc-site adsorption (Supplementary Fig. 12). The adsorption energies Δ*E* were calculated from the dipole corrected slabs with reference to gas-phase atoms. Spin-unrestricted calculations were performed for all SAA slabs, but for better convergence the charge density of a spin-restricted calculation was calculated first and used as starting guess for the spin-unrestricted calculation. Adsorption energies of CO and NO were calculated with the RPBE functional (see Supplementary Fig. 13 for comparison with optB86b-vdW)^38^.

Analysis of the electronic structure, that is, COHP analysis and pDOS, was performed using Lobster^26,39–42^. Starting from a VASP calculation with a higher 13×13×1 Monkhorst–Pack *k*-point mesh and symmetry switched off (ISYM = −1) the WAVECAR and other VASP output files where postprocessed using Lobster. Bader charges were determined using the VTST tools developed previously^43^.

The electronic population *n*_*j*_ of MO *j* can be determined summing over pDOS_*i*_ of its contributing AOs *i*. For bonding MOs, the integration over the energy 𝜖 is performed up to *E*^±^, the point where the COHP changes signs, that is, the point where the linear combination of AOs switches from being bonding to being antibonding (equation (1)), or up to the Fermi level *E*_F_ if *E*_F_ < *E*^±^. For antibonding MOs, the integration is performed from *E*^±^ up to *E*_F_ (equation (2)). If *E*^±^ > *E*_F_, the antibonding MO is empty (null population). Similar integrations are performed for the nonbonding *d* and *n*_*σ*_ orbitals (equations (3) and (4)).

1 $${n}_{j}{\rm{(bonding)}}=\sum _{i}{\int }_{-\infty }^{{{\min }}({E}_{\rm{F}},{E}_{i}^{\pm })}{\rm{pDOS}}_{i}({\epsilon }){\rm{d}}{\epsilon }$$

2 $${n}_{j}{\rm{(anti)}}=\sum _{i}{\int }_{{E}_{i}^{\pm }}^{{E}_{\rm{F}}}{\rm{pDOS}}_{i}({\epsilon }){\rm{d}}{\epsilon }$$

3 $${n}_{j}=\sum _{i}{\int }_{-\infty }^{{E}_{\rm{F}}}{\rm{pDOS}}_{i}({\epsilon }){\rm{d}}{\epsilon }$$

4 $${n}_{j}=\sum _{i}{\int }_{-\infty }^{{E}_{\rm{F}}}{\rm{pDOS}}_{i}({\epsilon }){\rm{d}}{\epsilon }-{n}_{j}{\rm{(bonding)}}-{n}_{j}{\rm{(anti)}}$$

Figure 3d was obtained using the VMD software (available at http://www.ks.uiuc.edu/Research/vmd/)^44^.

## Online content

Any methods, additional references, Nature Portfolio reporting summaries, source data, extended data, supplementary information, acknowledgements, peer review information; details of author contributions and competing interests; and statements of data and code availability are available at 10.1038/s41557-023-01424-6.

### Supplementary information

Supplementary Information Supplementary Figs. 1–13, Tables 1–3 and discussion.

### Source data

Source Data Fig. 1 Binding energies for each material.

Source Data Fig. 2 DOS and orbital filling.

Source Data Fig. 3 Adsorption energies for CO, NO, H_2_O and NH_3_; atomic charges for each material.

Source Data Fig. 4 Formation energies for N_2_ + H and NNH.

## Data Availability

The DFT calculations dataset used in this study is publicly available in the NOMAD Repository (10.17172/NOMAD/2023.12.04-2). Source data are provided with this paper.
