# Supplementary material for: Ten-electron count rule for the binding of adsorbates on single-atom alloy catalysts
Source: Nat Chem. 2024 Jan 23;16(5):749–54. doi: 10.1038/s41557-023-01424-6 (PMC11087240; doi:10.1038/s41557-023-01424-6)
Supplement: Supplementary file 1 — Supplementary Figs. 1–13, Tables 1–3 and discussion. [file 41557_2023_1424_MOESM1_ESM.pdf]

nature chemistry

Article

https://doi.org/10.1038/s41557-023-01424-6

Ten-electron count rule for the binding of adsorbates on single-atom alloy catalysts

In the format provided by the authors and unedited

Table of Contents

1. Comparison of the binding trends on pure transition metal surfaces and transition metal complexes. 1

2. Adsorption energies on Cu-, Ag-, and Au-based SAA 2

3. Comparison between the centres of the d-states of Ag-based SAAs with the d-band centres of pure transition metal surfaces 3

4. Adsorption energy for spin non-polarised 3d doped surfaces. 4

5. Construction of the Molecular Orbital diagrams 4

6. Electronic population analysis for clean surfaces and N adsorbed on 3d-doped Ag surfaces. 6

7. Role of the bonding and nonbonding orbitals 7

8. Bader charges of SAAs 8

9. Decomposition of the adsorption energy of H_2O and NH_3 8

10. Adsorption energy trends for halogens and hydroxyl OH 9

11. NNH geometry discussion 10

12. k-points dependence 11

13. Comparison of the adsorption site: N- and C-adsorption on atop and fcc sites 11

14. Comparison of different functionals 12

References 13

1. Comparison of the binding trends on pure transition metal surfaces and transition metal complexes.

On pure transition metals, the binding energy of adsorbates correlates linear with the centre of the *d*-band relative to the Fermi level (Supplementary Fig. 1a-b). Because of the discrete nature of electronic states on molecular complex, the monotonic trends break down. Approaching six ligands to a transition-metal atom (Supplementary Fig. 1d) results in the splitting of its *d*-orbitals into two degenerate e_g* antibonding orbitals and three degenerate t_{2g} orbitals, which are usually nonbonding but can gain a bonding or antibonding character when interacting with π-acceptor or π-donor ligands respectively.[1] This leads to non-monotonic trends in complex stability, which is quantified using the crystal field stabilisation energy (Supplementary Fig. 1c-e). Depending on the splitting between orbitals, the Crystal Field Stabilisation Energy (CFSE) shows as a deep V-shape

curve (low spin configurations) or a shallow W-shape curve (high spin configurations) when plotted against the number of d-electrons. Low spin complexes with six d-electrons are particularly stable: this corresponds to the saturation of the t_{2g} orbitals. When considering the two valence electrons that each ligand usually brings to the complex, the total number of valence electrons reaches 18-electrons.

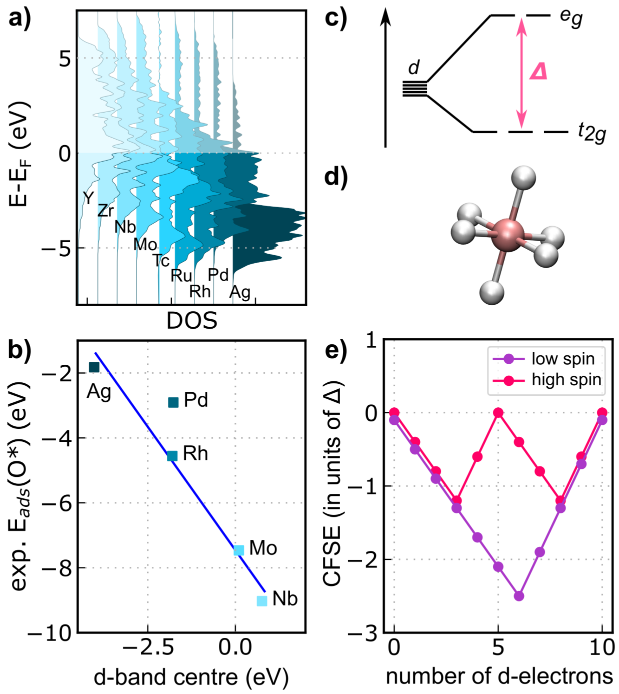

Supplementary Figure 1. Electronic properties controlling the binding energies of adsorbates on metal surfaces (a-b) and the binding energies of ligands in metal complexes (c-e). (a) Density of states (DOS) of *4d* metals. (b) Experimental adsorption energy of O adatoms as a function of the d-band centre for *4d* metals.[2,3] (c) Splitting of the degenerate *d*-orbitals into the t_{2g} and e_g* orbitals in octahedral complexes (represented in (d)). Δ is the energy difference between the two sets of orbitals. (e) Crystal field stabilisation energy (CFSE) plotted as a function of the number of *d*-electrons for low-spin and high-spin octahedral complexes. High-spin configurations are typically more common for 3d metals.

2. Adsorption energies on Cu-, Ag-, and Au-based SAA

Adsorption energies on the top site of the SAA dopant follow trends independent of the host metal. For clarity, we only show the adsorption energies on Au-based SAAs in Fig. 1 in the main manuscript. Supplementary Fig. 2 shows the complete set of transition metal dopants in Cu-, Ag-, and Au-hosts. For all hosts, *3d*-dopants show the shallow W-shape trend while *4d* and *5d* dopant show deeper V-shape trends. Because of the larger spatial expansion of *5d* orbitals, *5d* dopants tend to bind adsorbates more strongly than *4d* dopants. Substituting *4d* for *5d* dopants and vice versa can be used to finely tune the affinity of the dopant for a given substrate: we may want to increase the interaction for poorly reactive molecules or decrease the interaction in the presence of molecules that might poison the active site.

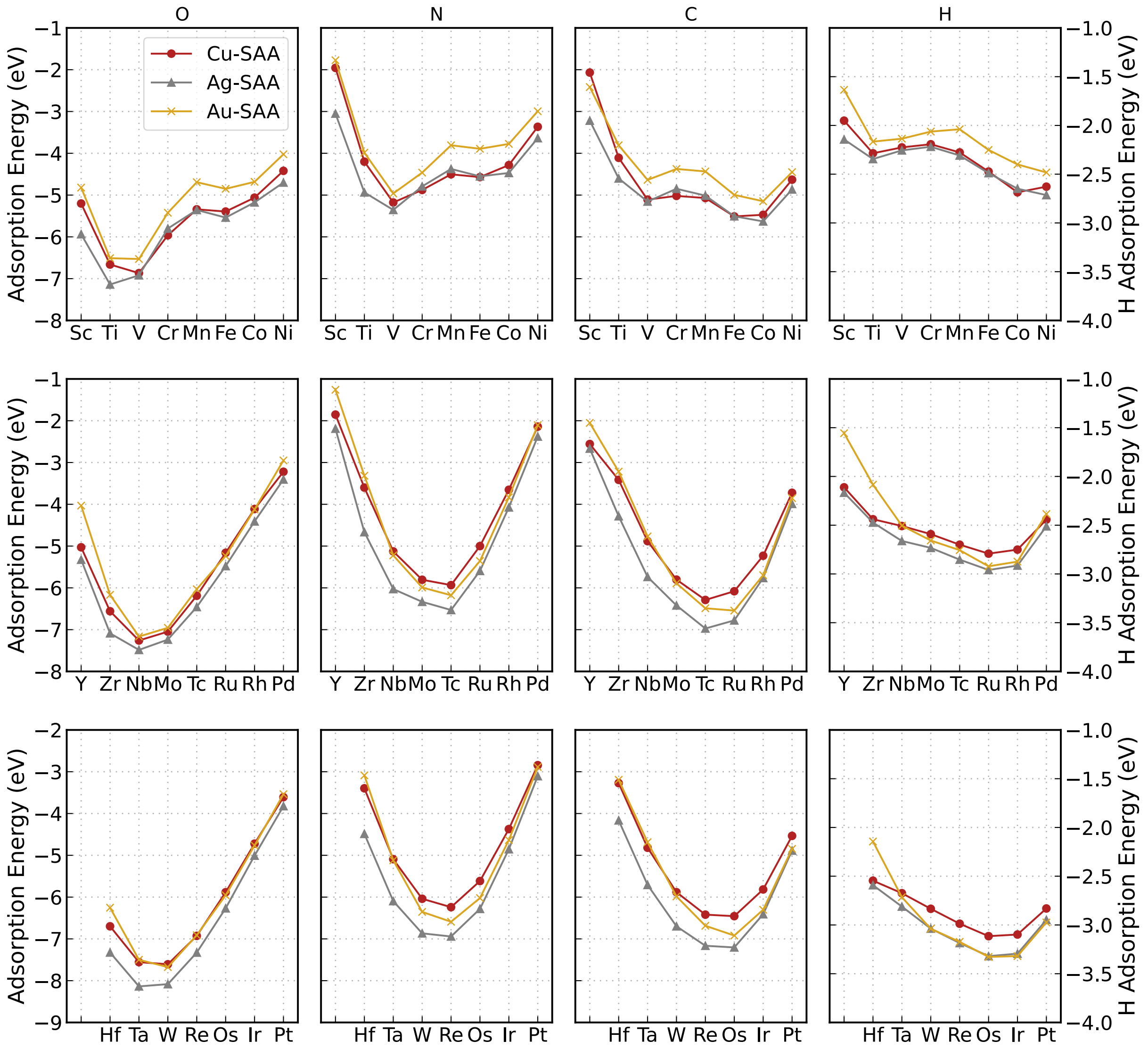

Supplementary Figure 2. Periodic trends for the binding energies of atomic adsorbates (O, N, C, H) on Cu-, Ag- and Au-based SAA surfaces doped with *3d* dopants (top row), *4d* (middle row), and *5d* (bottom row) dopants.

3. Comparison between the centres of the d-states of Ag-based SAAs with the *d*-band centres of pure transition metal surfaces

Supplementary Fig. 3 shows the comparison of pure and SAA dopant *d*-states. Pure transition metal surfaces' *d*-band centres and the centres of d-states of SAA dopants follow similar trends, as seen in Supplementary Fig. 3a. The *d*-band of pure surfaces (Supplementary Fig. 3b) is wider than the gas-phase like dopant states (Supplementary Fig. 3c).

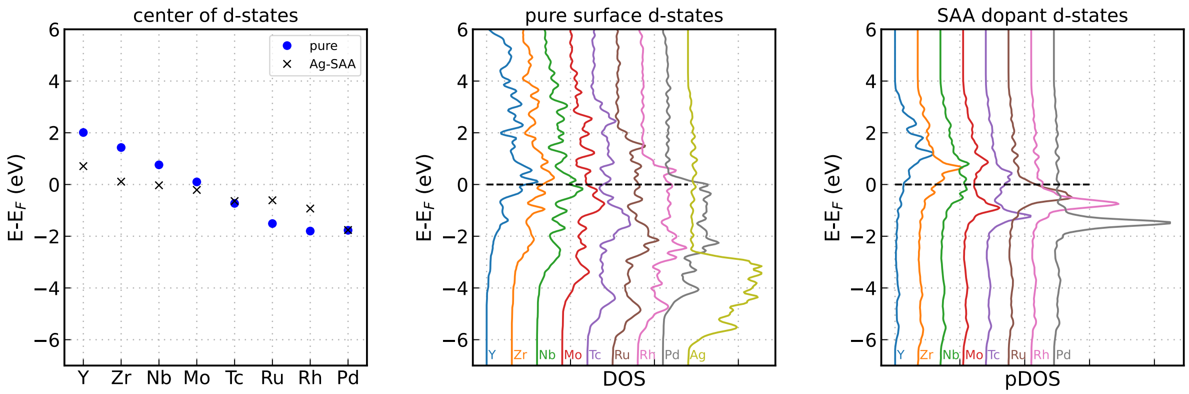

Supplementary Figure 3. Periodic trends in the *d*-band centres of Ag surfaces doped with 4d elements and the pure 4d transition metals. (a) Comparison of the d-band centres. (b) Density of states (DOS) of the pure transition metals. (c) SAA DOS projected (pDOS) on the states of the dopants.

4. Adsorption energy for spin non-polarised *3d* doped surfaces.

By performing spin restricted DFT, we can exclude spin effects. These calculations show that *3d*-dopants follow the same electron count rule as *4d* and *5d* dopants (see Supplementary Fig. 4). However, as the magnetic moment is quenched upon adsorption, spin effects destabilise the system. This destabilisation is strongest for the mid transition metal dopants Cr and Mn, which also possess the highest magnetic moments. That results in the altered W-shape of the curves (Fig. 1 and Supplementary Fig. 1).

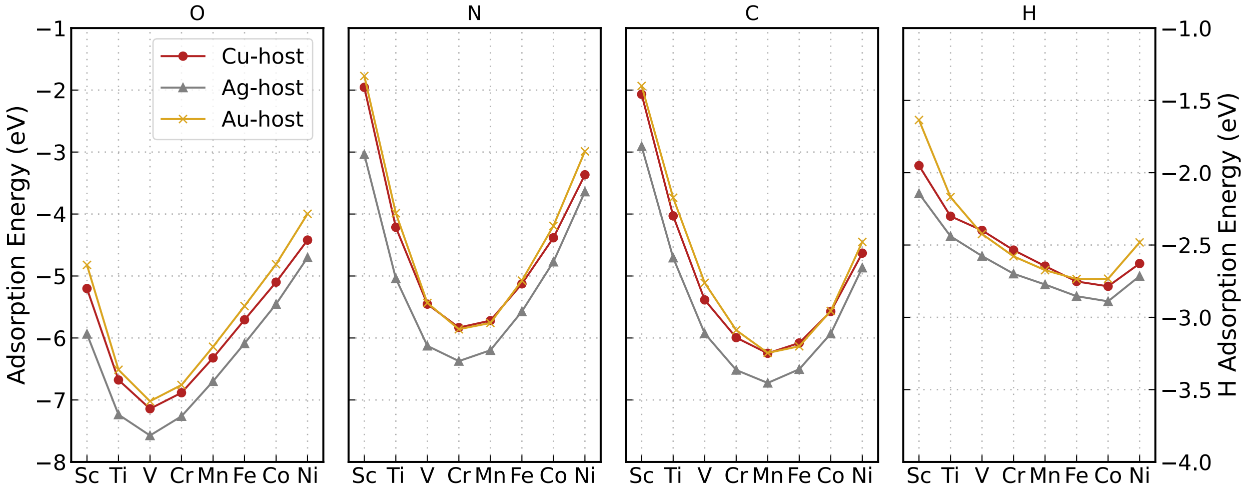

Supplementary Figure 4. Adsorption energies as calculated from spin non-polarised DFT calculations on 3d doped SAA surfaces.

5. Construction of the Molecular Orbital diagrams

If we ignore the host material, the M-A dinuclear complex (with M a transition metal, and A an adatom) is in the $C_{\infty v}$ point group. In this group, the irreducible representations of the *d*-orbitals are a_1 (d_{z^2}), e_1 (d_{xz}, d_{yz}) and e_2 (d_{xy}, $d_{x^2-y^2}$). For the hydrogen, the irreducible representation of the *1s*

orbital is a_1. This means, that only the *1s* orbital of hydrogen and the d_{z^2} orbital of the dopant have the right symmetry to interact and form a bonding σ and an antibonding σ^* molecular orbital (MO) as shown in Supplementary Fig. 5. The four other *d*-orbitals do not have suitable symmetry to form linear combinations; hence they form non-bonding n_δ and n_π MOs. This means we can fill up to five MOs (with ten electrons) before the antibonding σ^* orbitals get populated.

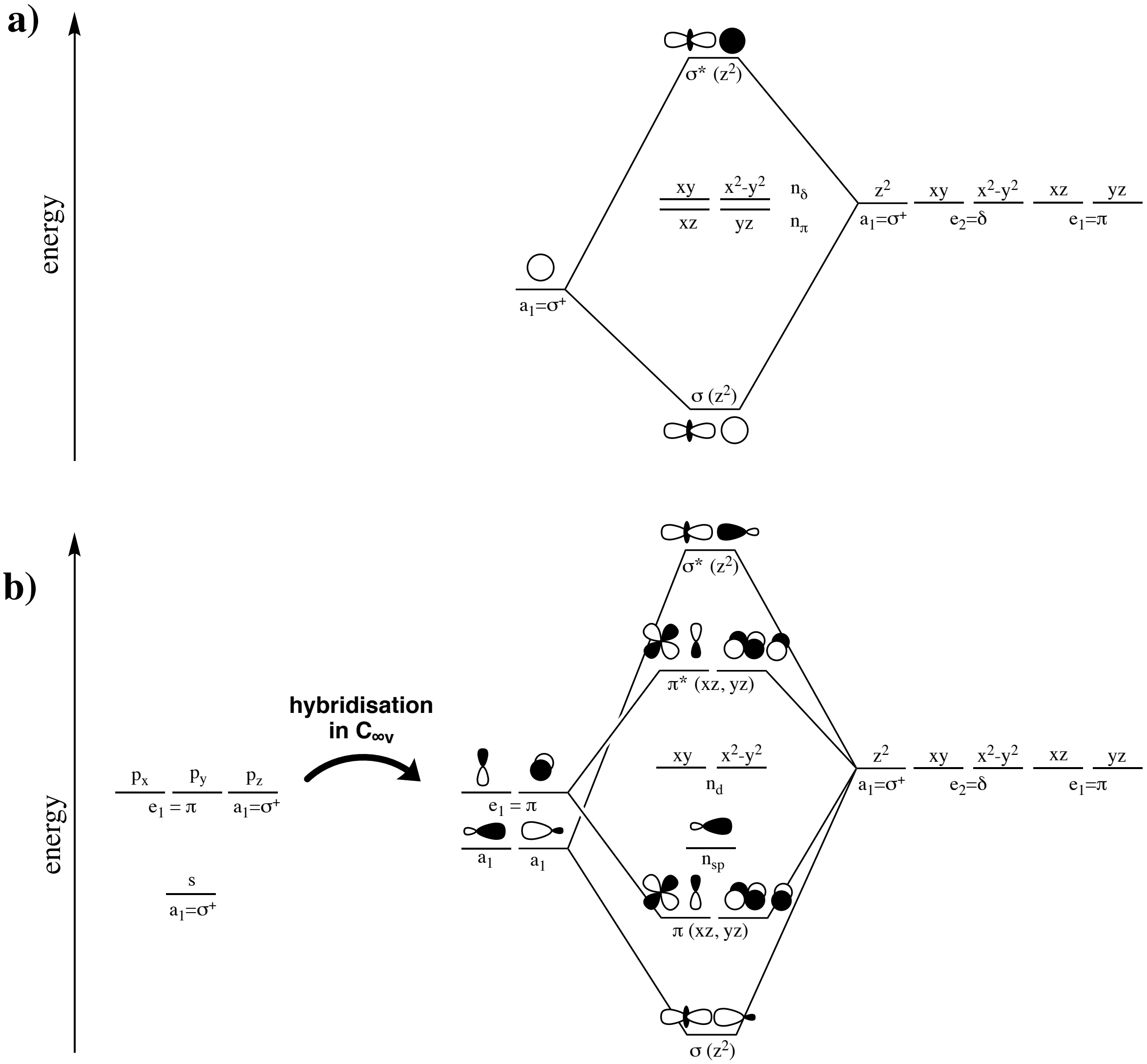

Supplementary Figure 5. Molecular Orbital diagram for the interaction of the *d*-orbital of a metal M with the orbitals of (a) hydrogen, and (b) *p*-block elements. The labels refer to the irreducible representation to which each orbital belongs in the $C_{\infty v}$ symmetry group.

For the adsorbates with *p*-electrons (C, N and O), the irreducible representations of the valence orbitals (*2s* and 2p) are a_1 (s, p_z) and e_1 (p_x, p_y). This means that the d_{z^2} orbital of the dopant can interact with both the *s* and p_z orbitals of the adatom, and thus, the three resulting MOs have a certain contribution of both the *s* and p_z orbitals. Pre-hybridising the *s* and p_z orbital to form two *sp* orbitals is an easy qualitative way to account for this mixing. One of the two resulting *sp* orbitals (Supplementary Fig. 5b) has a small lobe in the internuclear region, thereby poorly interacting with the dopant orbitals. This is the nonbonding n_{sp} orbital. The second *sp* orbital can form a bonding and antibonding orbital with the d_{z^2} orbital of the dopant (Supplementary Fig. 5b).

Similarly, the linear combination of d_{xz} d_{yz} with the p_x and p_y, forms two bonding π and two antibonding π* MOs. The d_{xy} and $d_{x^2-y^2}$ orbitals do not have the suitable symmetry to form a linear combination with any of the orbitals of the adatom, hence they form non-bonding n_δ MOs. This means for adsorbates with *p* orbitals, we can fill up to 6 MOs (with 12 electrons) before antibonding orbitals get populated.

For more than 10 or 12 electrons for H or *p*-element adsorbates respectively, antibonding MOs get filled, thereby weakening the bond, and destabilizing the system.

6. Electronic population analysis for clean surfaces and N adsorbed on 3d-doped Ag surfaces.

Supplementary Table 1. Electronic population analysis (*s* and *d* states) for Ag-based SAAs.

dopant	Number of valence electrons (υ_{M})		*s*-filling	*d*-filling	slab magnetisation
	nominal	s+d (from DFT)			in *Bohr magneton*
Sc	3	2.2	0.7	1.5	0.0
Ti	4	3.7	1.0	2.7	1.7
V	5	4.7	0.9	3.8	3.5
Cr	6	5.9	1.0	4.9	4.6
Mn	7	6.6	1.2	5.4	4.6
Fe	8	7.8	1.2	6.7	3.2
Co	9	9.0	1.1	7.9	2.0
Ni	10	10.1	1.0	9.1	0.0
Y	3	2.8	1.0	1.7	
Zr	4	4.1	1.3	2.8	
Nb	5	5.3	1.1	4.2	2.1
Mo	6	6.1	1.0	5.2	3.5
Tc	7	7.1	1.0	6.1	3.0
Ru	8	8.6	1.0	7.7	
Rh	9	9.7	1.0	8.7	
Pd	10	10.5	0.9	9.6	
Hf	4	3.9	1.4	2.6	
Ta	5	5.2	1.4	3.8	
W	6	6.2	1.3	4.9	2.9
Re	7	7.4	1.3	6.0	2.6
Os	8	8.6	1.3	7.4	0.0
Ir	9	9.7	1.2	8.4	
Pt	10	10.7	1.3	9.3	

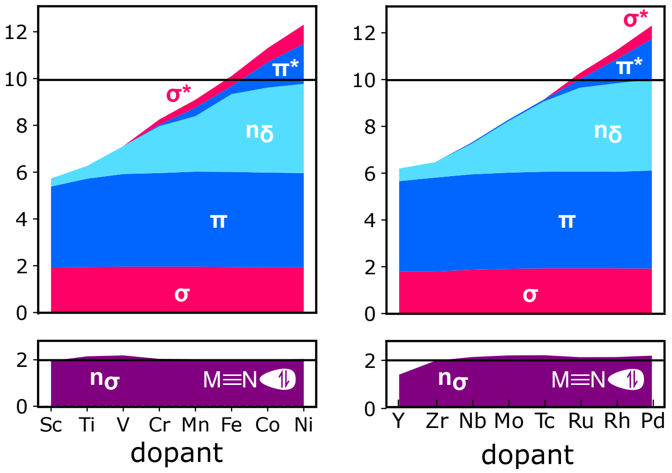

Supplementary Figure 6. Orbital occupation for N on Ag-based SAAs doped with *3d* (left panel) and *4d* (right panel) dopants. On 3d dopants the π^* and σ^* get filled earlier than on 4d dopants.

7. Role of the bonding and nonbonding orbitals

When going from left to right in the periodic table, dopants have more electrons. When the number of electrons exceeds a certain number, antibonding states start being populated: this weakens the adsorbate/dopant bond. But what drives the increased binding before that point? Nonbonding orbitals do not contribute to the stabilisation of the bond, so the origin of the stabilisation must be found elsewhere. Our calculations show that the dopant's states go down in energy as we move to the right of the periodic table. This allows the dopant's states to come closer (in energy) to the adsorbate's states. On top of this, the orbital contraction over a period allows adsorbates to come closer to the dopant, thereby strengthening the orbital interaction. This can be visualised by adding all the bonding and antibonding interactions up to the Fermi level. This is given by the ICOHP (integrated COHP signal) and the ICOOP (integrated crystal orbital overlap populations). They both show that the overlap (ICOOP) and overlap interaction (ICOHP) are extremal when the 10-electron criterion is met (Tc for N).

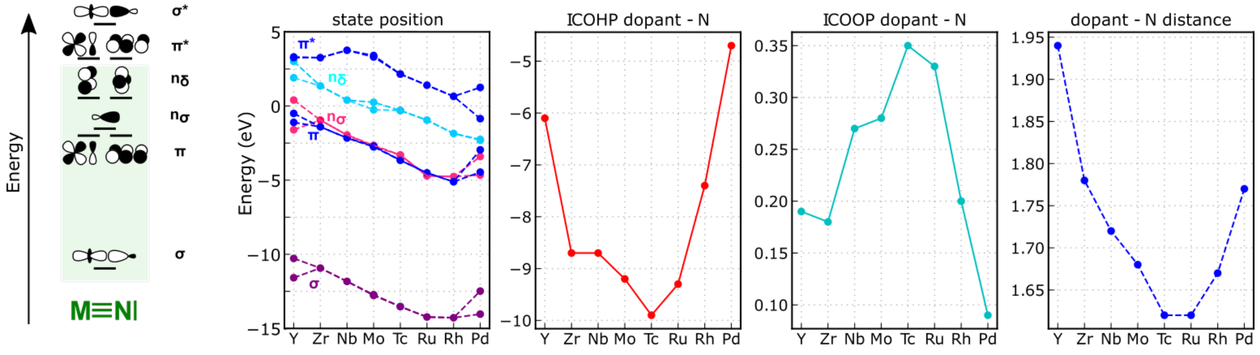

Supplementary Figure 7. Position of MOs for N-top adsorbed Ag-based SAAs as identified by the maximum/minimum of the COHP for bonding and anti-bonding states or the maximum of the pDOS of the respective non-bonding orbitals. Trends for integrated COHP (ICOHP), COOP and bond distances.

8. Bader charges of SAAs

Bader charges (Supplementary Table 2) arise from the electronegativity difference between the dopant and host metals. It is a good descriptor for the electrostatic contribution to bonding.

Supplementary Table 2. Bader charges of Cu-, Ag- and Au-based SAA.

DOPANT	CU-BASED SAA	AG-BASED SAA	AU-BASED SAA
**Sc**	1.41	1.47	1.65
**Ti**	1.08	1.10	1.38
**V**	0.79	0.83	1.11
**Cr**	0.59	0.65	0.92
**Mn**	0.52	0.66	0.94
**Fe**	0.26	0.43	0.76
**Co**	0.02	0.22	0.54
**Ni**	-0.10	0.05	0.34
**Y**	1.51	1.61	1.80
**Zr**	1.31	1.41	1.79
**Nb**	0.85	0.93	1.40
**Mo**	0.37	0.54	0.92
**Tc**	0.02	0.28	0.66
**Ru**	-0.22	-0.03	0.33
**Rh**	-0.35	-0.18	0.14
**Pd**	-0.36	-0.22	0.08
**Hf**	1.21	1.34	1.69
**Ta**	0.86	0.96	1.44
**W**	0.36	0.51	1.03
**Re**	-0.05	0.16	0.62
**Os**	-0.38	-0.17	0.26
**Ir**	-0.57	-0.37	0.01
**Pt**	-0.63	-0.46	-0.11

9. Decomposition of the adsorption energy of H_2O and NH_3

Following the approach developed by Réocreux *et al.*,[4] we can decompose the interaction energy into two terms:

- an electrostatic contribution that varies linearly with the atomic charge of the dopant q_d,
- a covalent contribution that varies linearly as a function of the binding energy of carbon E_{ads}^{C}.

The adsorption energy E_{ads} of H_2O or NH_3 can then be written as a linear combination of q_d and E_{ads}^{C}:

$$E_{ads} = \alpha + \beta q_d + \gamma E_{ads}^{C} \qquad \text{(i)}$$

Fitting the DFT computed adsorption energies on *4d*-doped Ag surfaces against the linear model gives the regression parameters provided in Supplementary Table 3.

Supplementary Table 3. Regression parameters for the linear model given in Eq. (i). MIN, MAX and STD stand for minimum, maximum and standard errors respectively.

	α (eV)	β (V)	γ	Min	Max	Std
H_2O	-0.175	0.456	0.085	-0.049	0.065	0.050
NH_3	-0.377	0.382	0.146	-0.071	0.071	0.064

In Supplementary Fig. 8, we plot the adsorption energies E_{ads}^{DFT} of H_2O (resp. NH_3) as computed with DFT (black curve), the electrostatic contribution βq_d (light blue curve) and the covalent contribution $E_{ads}^{DFT} - \beta q_d$ (red curve). The covalent contribution shows the expected trend with minima for d^8 dopants.

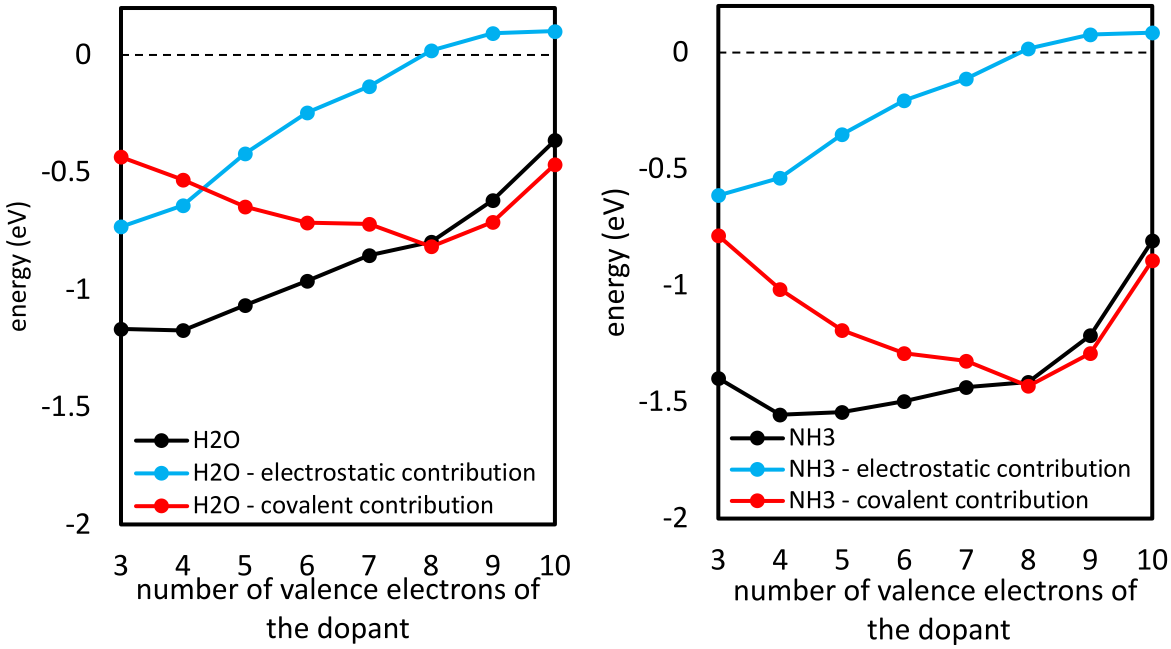

Supplementary Figure 8. Energy decomposition of the adsorption energy of H_2O and NH_3.

10. Adsorption energy trends for halogens and hydroxyl OH

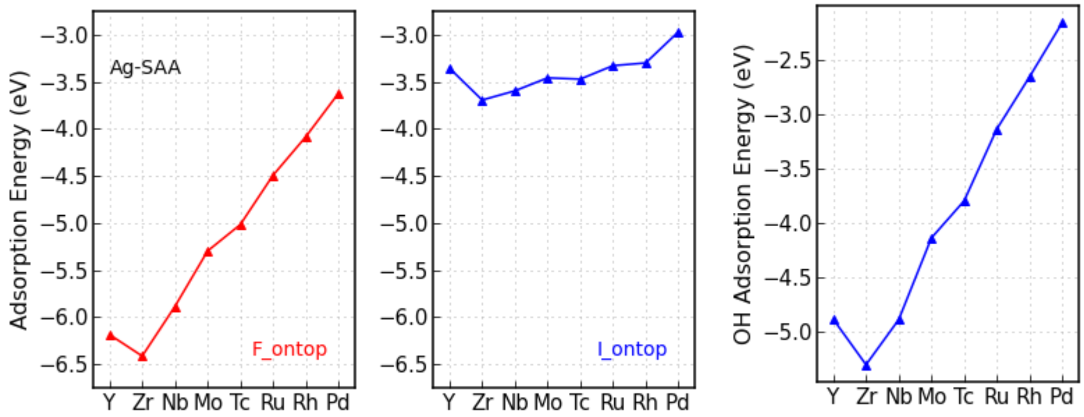

Supplementary Figure 9. Adsorption energy trends of fluorine, iodine and hydroxyl on Ag-SAA doped with *4d* metals.

11.NNH geometry discussion

In its linear geometry, usually considered on gold-based SAAs, NNH is isoelectronic to NO and therefore binds to the dopant with 3 electrons. On *3d* dopants, its binding energy shows the usual W-shape (Supplementary Fig. 19). On *4d* and *5d* dopants, we find a minimum for 7 electrons (Tc, Re), as expected from the 10-electron rule. Interestingly, we could not identify such geometry for the Pt and Pd dopants, which already have 10 electrons. With the three extra electrons from diazenyle, antibonding states would get populated. Instead, during the geometry optimisation, NNH relaxes to a bent geometry. The bent geometry allows for the hybridisation of the orbitals of the molecular fragment and transforms antibonding orbitals into non-bonding, located on the fragment, that can host these extra electrons (see lone pair in Fig. 4d). In this geometry, only one electron interacts directly with the dopant, with an expected minimum for d^9, hence its significance for late transition metals. For early transition metal dopants, a third configuration can be identified: the flat-lying geometry (Fig. 4d). To satisfy the 10-electron rule, the early transition metals try to create multiple bonds to get as many electrons as possible. In the flat-lying geometries, 5 electrons are available (Fig. 4d). DFT calculations confirm the highest stability for dopants with *ca*. 5 electrons (Nb, Ta, W) and such geometry is the most stable up for dopants up to 4 *d*-electrons (Supplementary Fig. 10).

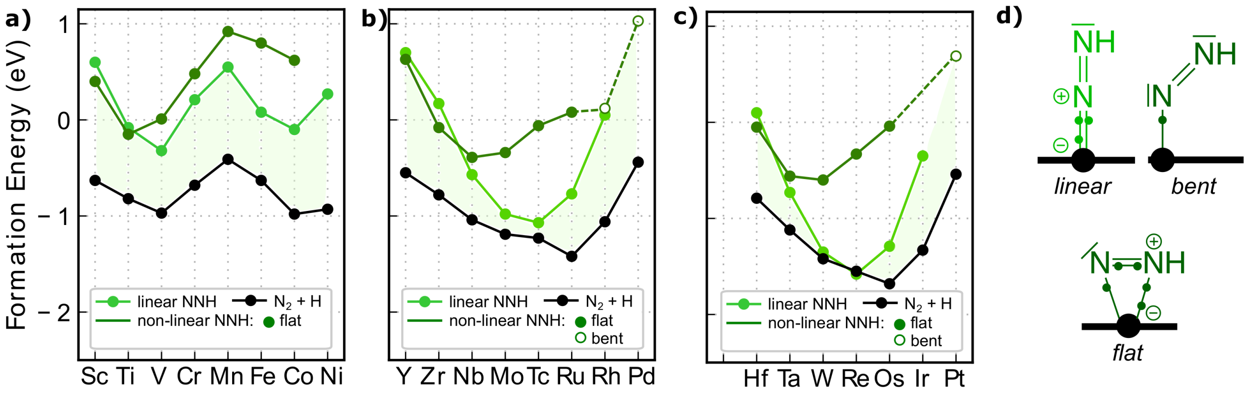

Supplementary Figure 10. Formation energies of NNH in the linear, flat, and bent geometries compared to that of N_2 on Au-based SAAs.

Regarding the relative stability of the different configurations, we can first notice the following points:

- flat-lying NNH is π-bonded to the dopant,
- bent NNH is σ-bonded to the dopant,
- linear NNH is σ and π-bonded to the dopant.

Because π -bonding is less effective than σ-bonding, the flat-lying geometry is expected to be less stable than the other two geometries. This is consistent with the general trend from our DFT calculations. A few exceptions are found for electron-deficient dopants (d^3 and d^4) for which flat-lying NNH and linear NNH show very similar stability (within DFT error). Finally, linear NNH is usually more stable than bent NNH because of the extra π-overlap. This holds true until the π* orbitals become occupied: then, the bent geometry becomes more favourable as explained in the previous paragraph. This is completely analogous to the configurational effects seen on metal complexes with the isolobal nitrosyl ligand and rationalised by Enemark and Feltham.[5]

12. *k*-points dependence

A relatively small *k*-point mesh (3×3×1) was used for the calculation of adsorption energies in this study. Supplementary Fig. 11 shows that a denser 13×13×1 *k*-point mesh results in the same trend with very minor differences to the adsorption energies.

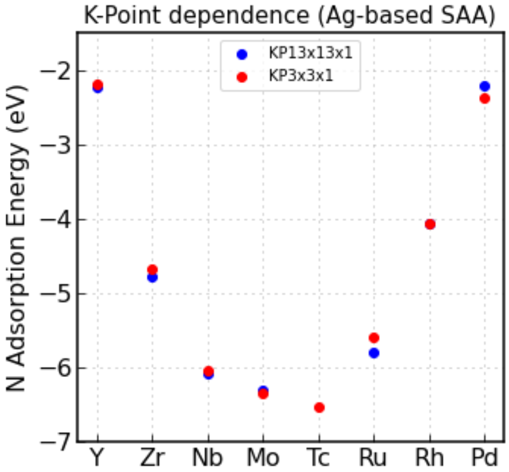

Supplementary Figure 11. Comparison of the adsorption energies of nitrogen adatoms for two *k*-point meshes: 3×3×1 vs 13×13×1.

13. Comparison of the adsorption site: N- and C-adsorption on atop and fcc sites

For this study, as adsorption site we used the atop position for all adsorbates. Even though this is not the most stable adsorption site for most adsorbates, we can show that the trends remain consistent. Supplementary Fig. 12 shows the N and C adsorption energies of atop and fcc adsorption on Cu-, Ag- and Au-based SAA dopants. The Cu host can compensate for the weaker adsorption on early and very late transition metal dopants, leading to a shallower adsorption energy trend of the fcc adsorption compared to atop adsorption.

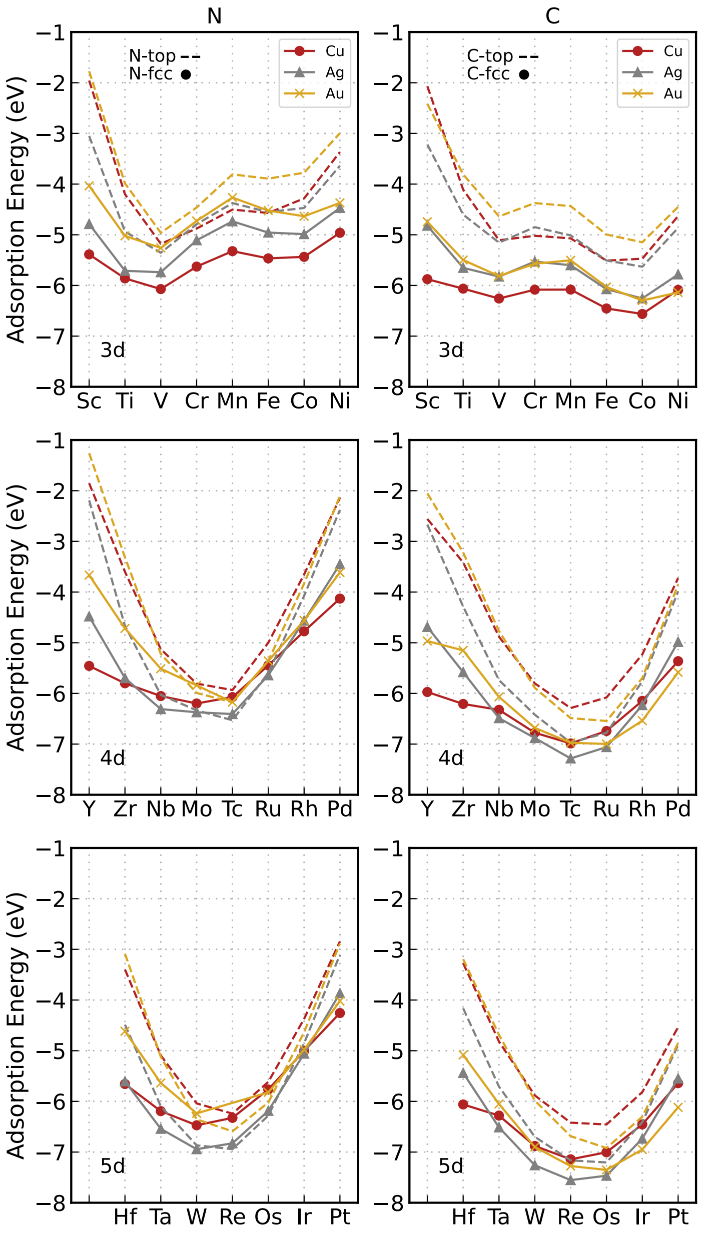

Supplementary Figure 12. Adsorption energies of nitrogen and carbon adatoms on Cu, Ag and Au-based SAA surfaces at the atop (dashed lines) and fcc hollow (solid lines with markers) adsorption sites.

14. Comparison of different functionals

The characteristic W and V-shape for the adsorption energy of covalently bonded atomic and molecular adsorbate is reproduced independent of the functional. Supplementary Fig. 13 shows the comparison of the optB86b-vdW and well-known RPBE functional.

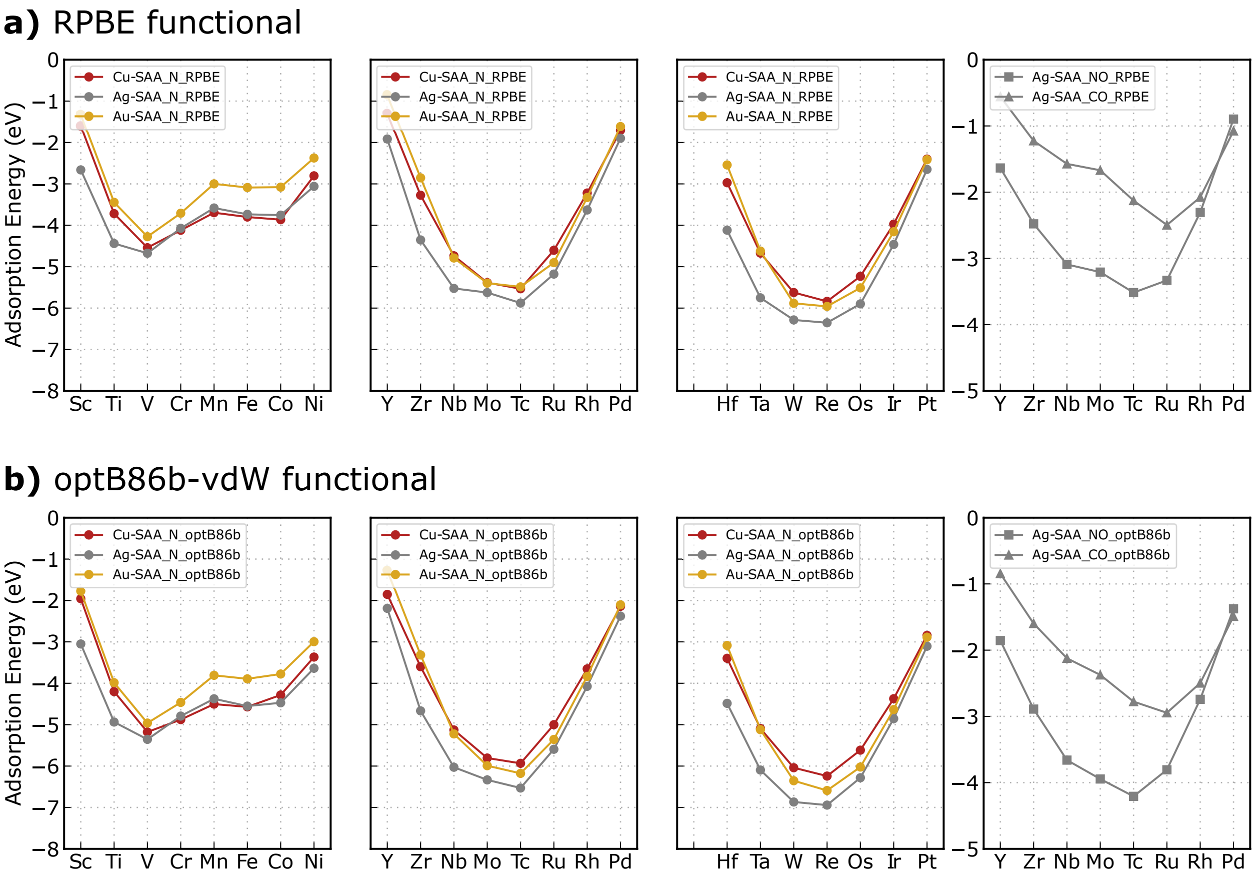

Supplementary Figure 13. Adsorption energies of N, CO and NO calculated with 2 different DFT functionals, RPBE and optB86b-vdW. Both functionals give the same trends.

References

1. Housecroft, C. E. & Sharpe, A. G. *Inorganic Chemistry*. (Pearson Education, 2012).
2. Lawless, K. R. The oxidation of metals. *Reports on Progress in Physics* **37**, 231–316 (1974).
3. Toyoshima, I. & Somorjai, G. A. Heats of Chemisorption of O2 , H2 , CO, CO2 , and N2 on Polycrystalline and Single Crystal Transition Metal Surfaces. *Catalysis Reviews* **19**, 105–159 (1979).
4. Réocreux, R., Sykes, E. C. H., Michaelides, A. & Stamatakis, M. Stick or Spill? Scaling Relationships for the Binding Energies of Adsorbates on Single-Atom Alloy Catalysts. *J Phys Chem Lett* **13**, 7314–7319 (2022).
5. Enemark, J. H. & Feltham, R. D. Principles of structure, bonding, and reactivity for metal nitrosyl complexes. *Coord Chem Rev* **13**, 339–406 (1974).
